# The mediation effect analysis of nurse’s mental health status and burnout under COVID-19 epidemic

**DOI:** 10.3389/fpubh.2023.1221501

**Published:** 2023-10-17

**Authors:** Fuzhi Liu, Yanyan Zhao, Yangjia Chen, Zhuote Tu

**Affiliations:** ^1^Department of Preventive Medicine, School of Health, Quanzhou Medical College, Quanzhou, China; ^2^Department of Nursing, Quanzhou First Hospital, Quanzhou, China

**Keywords:** nurse, anxiety, depression, burnout, occupational health, structural equation model

## Abstract

**Aim:**

The objective of this study is to investigate the mental health status of nurses during the outbreak of novel coronavirus pneumonia. Additionally, we aim to analyze the relationship between anxiety, depression, and burnout among nurses. The findings will provide a scientific basis for promoting the psychological health of nurses.

**Methods:**

Using a cross-sectional study, nurses in Quanzhou in May 2020 completed a general information questionnaire, the 7-item Generalized Anxiety Disorder Questionnaire (GAD-7), Patient Health Questionnaire-9 (PHQ-9), and the Maslach Burnout Inventory (MBI). Data analysis was conducted using structural equation model.

**Results:**

372 questionnaires were returned, with a response rate of 92.5%. The prevalence of anxiety and depression among the participants were 45.2 and 41.4%, respectively. The prevalence of severe burnout among nurses was found to be 7.3%. There was a correlation between nurses’ anxiety, depression, and job burnout. The correlation coefficients between anxiety and job burnout, depression and job burnout, and anxiety and depression were found to be statistically significant (*p* < 0.001). Depression plays a mediating role between anxiety and jod burnout (0.584/1.413, 41.3%).

**Conclusion:**

The COVID-19 epidemic has resulted in moderate to high levels of job burnout among nurses. In this context, depression has been found to play a mediating role in the relationship between anxiety and job burnout. It is imperative for hospital administrators to prioritize the mental health of nurses and the provide necessary support to ensure their well-being.

## 1. Introduction

Nurses are increasingly being acknowledged for their significant contributions in the areas of primary care, public health emergencies, chronic disease management, surveillance, and the identification of new and emerging infectious diseases (1, 2). Four distinct roles for nurses in primary care and public health collaboration have been identified: relationship builder, outreach professional, program facilitator, and care coordinator (3). The prolonged engagement in these medical tasks will have an impact on the physical and mental health of nurses.

Nurses are a significant group within the medical field, comprising the largest proportion of personnel in various medical institutions at all levels. Research conducted in the past has demonstrated a correlation between nurse burnout and negative mental health outcomes, specifically depression and anxiety (4, 5). Numerous studies have demonstrated that public health emergencies, including infectious disease outbreaks and natural disasters, can significantly impact the physical and mental well-being of nurses, leading to job burnout (6, 7). In addition to their daily workload, nurses are also at the forefront of preventing and controlling the spread of the new coronavirus in a unique work environment. As a result, their workload has significantly increased. The scale and duration of the COVID-19 pandemic is larger and longer compared to previous infectious disease epidemics. As a result, the impact on the mental health of nurses has been more significant and widespread (8, 9). Prolonged exposure to high levels of stress can cause nurses to experience emotional, attitudinal, and behavioral exhaustion, ultimately resulting in job burnout.

The concept of burnout was first introduced by Freudenberger in 1974 (10), who believed that long-term exposure to interpersonal stressors at work caused a state of physical and mental exhaustion related to nursing activities. Later, Maslach identified the psychological syndrome caused by long-term emotional and interpersonal stressors at work as job burnout, which is characterized by emotional exhaustion, depersonalization, and decreased personal accomplishment (11). This study employed the aforementioned concept to establish a comprehensive definition of burnout within the nursing profession.

This study aims to investigate the current state of job burnout among nurses in Quanzhou City during the COVID-19 epidemic, as well as the factors that contribute to it. The study hypothesizes that there is a close relationship between anxiety, depression and job burnout, and utilizes a structural equation model to explore the relationship between the three variables. The findings of this study will provide valuable data to help alleviate nurses’ job burnout, maintain their mental health, and increase their motivation to work.

## 2. Theoretical background

There is a body of research that has focused extensively on COVID-19 pandemic affected the mental health of nurses. Mental problems related to the health emergency, such as stress, anxiety, depression, traumatic distress response, post-traumatic stress disorder (PTSD), and sleep disorders are more likely to affect medical and nursing staff (12–15). The first paper carried on the mental health of 994 healthcare workers in Wuhan, and the results indicated that 36.9% had subthreshold mental health disturbances, 34.4% had mild disturbances, 22.4% had moderate disturbances and 6.2% had severe disturbance (16). A systematic review and meta-analysis, which included in the analysis with a combined total of 33,062 participants from thirteen studies, showed that anxiety with a pooled prevalence of 23.2%, depression with a prevalence rate of 22.8% and insomnia with a prevalence rate of 38.9% (17). Another literature was reviewed for mental health problems of healthcare workers during the COVID-19 pandemic. The findings reported that pooled prevalence of mental health problems for post-traumatic stress disorder, anxiety, depression, and distress was 49, 40, 37, and 37%, respectively (18).

Amidst the COVID-19 pandemic, nurses find themselves susceptible to experiencing anxiety and depression due to the numerous psychological stressors they encounter. Consequently, managing and coping with these mental health challenges has become a prevalent issue worldwide (19). The current literature on COVID-19 supports the idea that the increased workload during a pandemic can lead to higher levels of anxiety and depression among individuals, which in turn poses a significant threat to the overall well-being of nurses (20, 21). Anxiety and depression often occur together (22, 23), although the exact relationship between these two conditions is still uncertain (24). The temporal relationship between anxiety and depression is a topic that has been widely discussed and debated among researchers. Some researchers posit that anxiety serves as a precursor to depression, implying a unidirectional association (25, 26). On the contrary, there are proponents who suggest a bidirectional association between the two aforementioned conditions (27, 28). Nevertheless, it has been observed that symptoms of anxiety have a stronger predictive value for later depressive symptoms compared to the reverse relationship.

Burnout is often occurred in the nurse, which has negatively impacted the quality of care, patient safety, and the functioning of staff workers in the health care industry (29). Substantial systematic reviews and meta-analyses have already shown that the prevalence of burnout is different between regions and times (30, 31). Mental health is one of primary health outcomes of burnout, and it refers to the nurse’s mental health as an individual, the high rates of trauma, depression, stress, and anxiety seen in many nurses, and how poor mental health often leads to burnout and *vice-versa* (32). Due to, the pandemic of COVID-19 exacerbating the complexity of the work environment, the problem of burnout among nurses has become more pronounced. Anxiety and depression are the most common psychological problems that can easily lead to job burnout, as evidenced by many literatures (5, 33, 34). In addition, burnout is affected by many other factors such as psychological resilience (35, 36), fear (37, 38), and psychological inflexibility (39).

**Figure 1:**
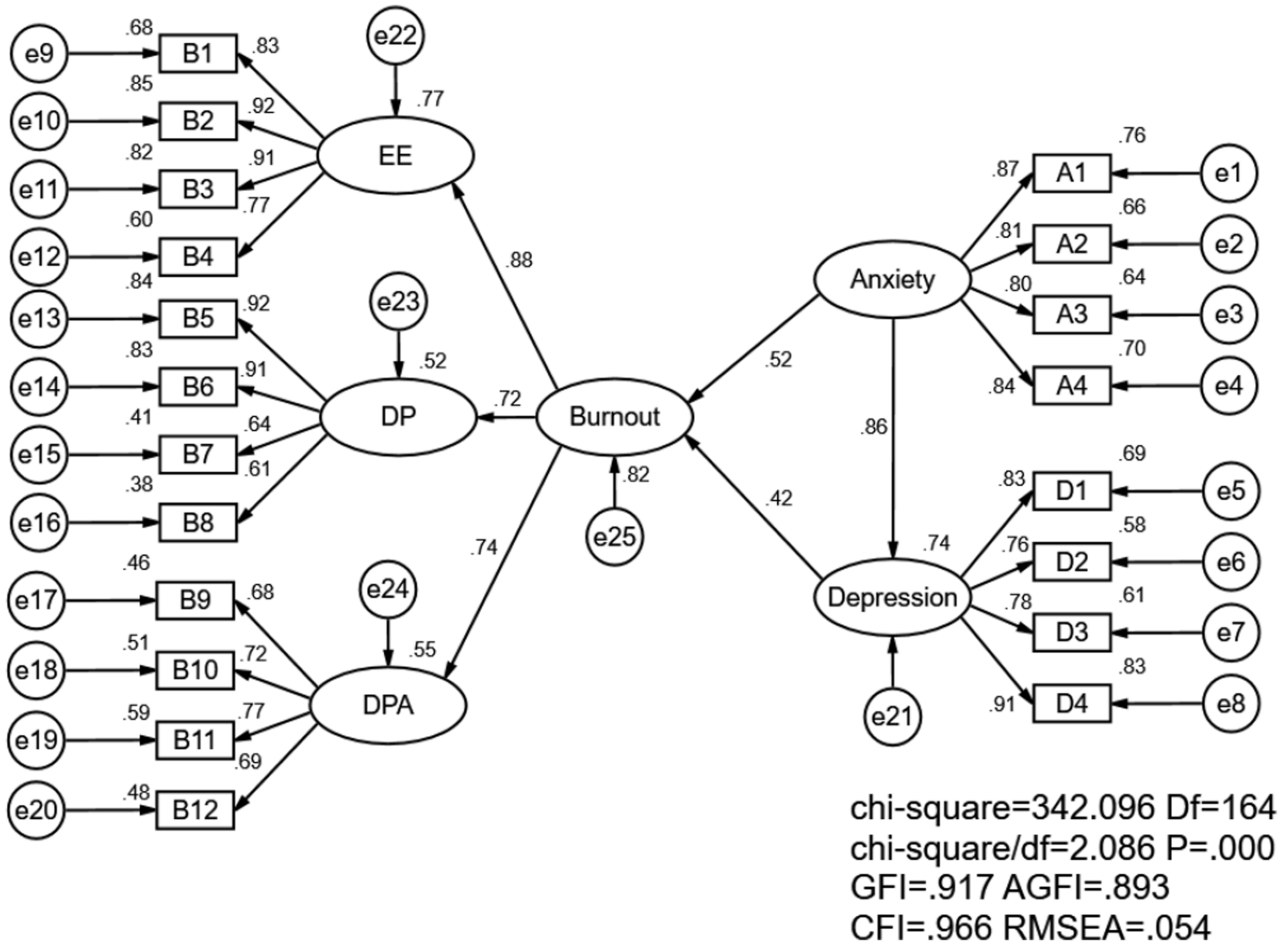
Structural equation model for nurse burnout, anxiety, and depression. EE indicate emotional exhaustion; DP indicate depersonalization and DPA indicate decreased personal accomplishment.

Burnout, depression, and anxiety are postulated to exhibit interrelationships. We aimed to test the mediating role of depression in the relationship between anxiety and burnout. Based on the above literature review, we propose the following model. From this model, we propose three main hypotheses: (see Figure 1)

*H1*: Anxiety has a positive and significant impact on job burnout.

*H2*: Depression has a positive and significant impact on job burnout.

*H3*: Depression mediate the relationship between anxiety and job burnout.

## 3. Materials and methods

### 3.1. Participants

The sample for this study included 372 nurses (96.5% females) whose ages ranged between 20 and 59 years, with a mean age of 30.8 ± 6.3 years. 64.8% of participants were married and 190 (51.1%) have a junior college degree. The majority of nurses (76.9%) have less than 10 years of work experience (Table 1).

**Table 1:** Characteristics of the sample.

Variables	Classification	*N*	%
Sex	Female	359	96.5
	Male	13	3.5
Marriage	Married	241	64.8
	Others	131	35.2
Education	High school	11	3.0
	College	190	51.1
	Bachelor or above degree	171	45.9
Work experience	﹤5 years	138	37.1
	5–10 years	148	39.8
	11–20 years	56	15.0
	﹥20 years	30	8.1

### 3.2. Measures

#### 3.2.1. General information questionnaire

A self-designed general situation questionnaire has been created based on literature research and expert opinions The questionnaire includes basic demographic information such as age, gender, and marital status.

#### 3.2.2. The 7-item generalized anxiety disorder questionnaire (GAD-7)

There are 9 items in the scale, and 4 grades are used for scoring (40). The scoring rules are as follows: 0–4 is normal, 5–9 is mild anxiety, 10–14 is moderate anxiety, and 15–21 is severe anxiety (41). In this study, Cronbach’s α coefficient = 0.943; KMO = 0.926, Bartlett sphericity test *p* < 0.01.

#### 3.2.3. Patient health questionnaire-9 (PHQ-9)

There are 9 items in the scale, and 4 grades are used for scoring (42). The scoring rules are as follows: 0–4 means no depression, 5–9 means mild depression, 10–14 means moderate depression, and 15–19 means moderate depression. Major depressive disorder, 20–27 may have major depressive disorder (43). In this study, Cronbach’s α coefficient = 0.922; KMO = 0.902, Bartlett sphericity test *p* < 0.01.

#### 3.2.4. Maslach burnout inventory (MBI)

This test includes three aspects of job burnout: the scale contains 3 dimensions, 22 items, and uses Likert 7-level scoring, scoring rules: 9 items of emotional exhaustion (EE), indicating the emotional response caused by work pressure, with a total score of 0–54. Scores ≤19, 20–25 and ≥ 26 represent low, medium and high level of EE respectively; 5 items of depersonalization (DP) indicate the attitudes and feelings of clients caused by work pressure, with a total score of 0–30 and scores ≤6, 7 ~ 8 and ≥ 9 represent low, medium, and high level of DP respectively; 8 items with decreased personal accomplishment (DPA) indicate negative self-evaluation, accompanied by a decline in work ability experience and achievement experience (11). The total score is 0 ~ 48 points, and the score is ≤34, 35 ~ 38, and ≥ 39 represent high, medium, and low level of DPA, respectively, and the scores are reversed. We operationalized high-level burnout as the presence of elevated levels in all three dimensions. Medium-level burnout was defined as elevated levels in two dimensions, while low-level burnout was characterized by elevated levels in only one dimension. The absence of burnout was indicated when all levels were low (44–46). In this study, Cronbach’s α coefficient = 0.906, KMO = 0.926, Bartlett sphericity test *p* < 0.01.

### 3.3. Procedures

A quantitative research design using a self-reported questionnaire was used to collect data from a tertiary designated hospital in Quanzhou, Fujian Province who were treating patients with new coronary pneumonia. The hospital has a total of 1,621 nurses, who are assigned to various wards, with a distribution of 67 wards. To gather data from nurses, the quota sampling method was employed, wherein 6 participants were randomly selected from each ward, resulting in a total sample size of 402.

The inclusion criteria were: (1) working for at least one year, (2) providing informed consent to participate in the study, and (3) working during the COVID-19 epidemic. The exclusion criteria were: (1) receiving psychological counseling or treatment in the past year, and (2) having a prior history of mental illness.

We designed the online questionnaire using the Wenjuanxing platform and sent the survey link to nurses *via* WeChat, QQ or email. We completed data collection from May 1st to 30th, 2020. The entire questionnaire took at least 5 min to complete, and samples with response times below this standard were removed. At the beginning of the questionnaire, there is an informed consent form, participants need to read this section and click on consent. We are also making a statement that the data from this survey will only be used for scientific research and will not disclose the personal information of the participants. This study was approved by the ethical committee of Quanzhou First Hospital (NO.Quan Yi lun 2,020,181).

### 3.4. Data analysis

Data cleaning, normality test and correlation analysis were performed using SPSS Version 26.0. A structural equation model was established using AMOS24.0 software to fit the relationship model between nurses’ anxiety, depression and job burnout. Following the suggestion of Gerbing and Anderson (47), we estimate the measurement model separately for each construct before constructing the model. In this study, the model was estimated by the maximum likelihood estimation, and the model was corrected with square multiple correlation (SMC) (48) and the modification index (MI). Since all aspects are reflective indicators, if the requirements of SMC and MI are not met during model modification, we will delete the question (49, 50). We has performed the Sobel test (51) and bootstrap method (52) for assessing the mediating effects. Five indices were employed to evaluate the adequacy of fit for both the measurement and structural models: Normed Chi-square (χ2/df < 3) (53, 54), Goodness of Fit Index (GFI > 0.90), Adjusted-Goodness of Fit Index (AGFI>0.90), Comparative Fit Index (CFI > 0.90) (55), Root Mean Square Error of Approximation (RMSEA<0.08) (56).

## 4. Results

### 4.1. Anxiety, depression and burnout among nurses based on demographic information

Burnout was statistically different in education and work experience, but the rest were not different (Table 2).

**Table 2:** Anxiety, depression and burnout among nurses based on demographic information.

Variables	Classification	*N*	Anxiety	Depression	Burnout
Sex	Female	359	4.12 ± 4.38	5.15 ± 5.68	58.50 ± 17.98
	Male	13	3.62 ± 3.33	3.62 ± 3.22	56.62 ± 15.25
	*t*		−0.411	−0.966	−0.372
	*p*		0.681	0.335	0.710
Marriage	Married	241	4.31 ± 4.49	5.20 ± 5.82	59.68 ± 18.59
	Others	131	3.73 ± 4.05	4.91 ± 5.24	56.14 ± 16.30
	*t*		−1.235	−0.470	−1.830
	*p*		0.218	0.639	0.068
Education	High school	11	5.36 ± 5.278	8.64 ± 7.032	59.10 ± 16.69
	College	190	3.73 ± 4.441	4.71 ± 5.671	55.72 ± 18.67
	Bachelor or above degree	171	4.43 ± 4.152	5.3 ± 5.404	61.40 ± 16.62
	*F*		1.657	2.781	4.654
	*p*		0.192	0.063	0.010^*^
Work experience	﹤5 years	138	3.62 ± 3.82	4.49 ± 5.02	55.22 ± 16.60
	5–10 years	148	4.37 ± 4.796	5.68 ± 5.952	59.18 ± 19.61
	11–20 years	56	4.07 ± 3.726	4.61 ± 4.979	60.34 ± 15.38
	﹥20 years	30	5.07 ± 5.199	5.9 ± 7.317	65.97 ± 16.40
	*F*		1.263	1.406	3.639
	*p*		0.287	0.241	0.013^*^

*Indicate *p* less than 0.05.

### 4.2. Anxiety, depression and burnout among nurses

The anxiety score was (4.10 ± 4.34), and the depression score was (5.09 ± 5.62). The prevalence of anxiety and depression among the participants were 45.2 and 41.4%, respectively.

The research conducted on the phenomenon of job burnout among nurses yielded noteworthy findings. Specifically, the average score for emotional exhaustion was determined to be 22.12, with a significant proportion of participants (59.4%) displaying medium to high levels of this particular dimension. Similarly, the average score for depersonalization was found to be 9.62, and a substantial majority of nurses (70.4%) exhibited moderate to high levels of this aspect. Additionally, the average score for decreased personal accomplishment was calculated to be 26.70, with 64.0% of participants falling within the medium to high range for this dimension. The prevalence of severe burnout among nurses was found to be 7.3% (Table 3).

**Table 3:** Classification and scores of nurse burnout.

Dimension	Low (*n*, %)	Moderate (*n*, %)	High (*n*, %)	x̅±s
Emotional exhaustion	151 (40.6)	122 (32.8)	99 (26.6)	22.12 ± 9.40
Depersonalization	110 (29.6)	102 (27.4)	160 (43.0)	9.62 ± 4.35
Decreased personal accomplishment	134 (36.0)	83 (22.3)	155 (41.7)	26.70 ± 9.70

### 4.3. Analysis of anxiety, depression and burnout among nurses

The pearson correlation was used to analyze the relationship between anxiety, depression and burnout among nurses, which showed a positive correlation between anxiety and depression on burnout (Table 4).

**Table 4:** Analysis of anxiety, depression and burnout among nurses.

	Anxiety	Depression	Burnout
Anxiety	1.000		
Depression	0.851^*^	1.000	
Burnout	0.639^*^	0.616^*^	1.000

^*^Indicate *p* less than 0.001.

### 4.4. Structural equation model of burnout, anxiety, and depression among nurses

#### 4.4.1. Selection of the model metrics

To assess whether the measurement indicators accurately reflected the latent variables, a Confirmatory Factor Analysis (CFA) was conducted on all constructs.

Out of the original 7 items in the anxiety questionnaire, only 4 items had standardized factor loadings above 0.7 (but not exceeding 0.95) and significant positive residuals after CFA. These 4 questions were deemed reliable with a Composite Reliability (CR) of 0.899 (exceeding the standard of 0.7) (57) and an Average Variance Extracted (AVE) of 0.690 (exceeding the standard of 0.5) (58). Additionally, the fitting index fell within the acceptable range, thus confirming the retention of these 4 questions.

Out of the 9 items in the depression questionnaire, only 4 had standardized factor loadings exceeding 0.7 but not exceeding 0.95, and all residuals were positive and significant after CFA. The CR was 0.892, surpassing the standard of 0.7, and the AVE was 0.677, surpassing the standard of 0.5. The fitting index was within the acceptable range, therefore, these 4 questions were retained.

In this study, job burnout was analyzed as a second-order aspect and deconstructed into three dimensions: emotional exhaustion, depersonalization, and decreased personal accomplishment. To ensure the validity of these dimensions, a first-order three-factor complete correlation model and a second-order factor model were analyzed. This study utilized the first-order three-factor complete correlation model to analyze the data. The results showed that the first-order factors had moderate to high correlations. Specifically, emotional exhaustion and job apathy had a correlation coefficient of 0.66, depersonalization and decreased personal accomplishment had a correlation coefficient of 0.52, and emotional exhaustion and decreased personal accomplishment had a correlation coefficient of 0.63. Additionally, the target coefficient was 100%, indicating that the second-order job burnout in this study met the requirements of the theoretical model. The residuals of the standardized factor loadings for the three deconstructed constructs were all found to be positive and significant after the second-order CFA. Additionally, the CR exceeded the standard of 0.7, the AVE exceeded the standard of 0.5, and the fitting index fell within the acceptable range. As a result, the second-order three-facet model was retained for further analysis (Tables 5, 6).

**Table 5:** The model estimation parameters and fit indices for different surfaces.

Surfaces	Indices	*Unstd.*	*S.E.*	*t*	*p*	*std.*	*SMC*	*CR*	*AVE*	*χ2*	*df*	*χ2/df*	*GFI*	*AGFI*	*RMSEA*
Anxiety	A1	1.000				0.887	0.787	0.899	0.690	0.817	2	0.409	0.999	0.994	0.000
	A2	0.894	0.047	19.146	^*^	0.806	0.650								
	A3	0.925	0.048	19.120	^*^	0.805	0.648								
	A4	0.998	0.051	19.729	^*^	0.822	0.676								
Depression	D1	1.000			^*^	0.824	0.679	0.892	0.677	4.342	2	2.171	0.994	0.970	0.056
	D2	1.021	0.059	17.422	^*^	0.786	0.618								
	D3	1.156	0.054	21.284	^*^	0.937	0.878								
	D4	0.772	0.049	15.681	^*^	0.729	0.531								
Emotional exhaustion	B1	1.000			^*^	0.808	0.653	0.917	0.736	5.085	2	2.543	0.993	0.996	0.064
	B2	1.384	0.063	22.060	^*^	0.941	0.885								
	B3	1.320	0.063	21.011	^*^	0.903	0.815								
	B4	1.095	0.066	16.701	^*^	0.768	0.590								
Depersonalization	B5	1.000			^*^	0.922	0.850	0.858	0.611	3.125	2	1.562	0.996	0.980	0.039
	B6	1.046	0.045	23.158	^*^	0.909	0.826								
	B7	0.602	0.043	14.075	^*^	0.640	0.410								
	B8	0.676	0.053	12.847	^*^	0.599	0.359								
Decreased personal accomplishment	B9	1			^*^	0.677	0.458	0.806	0.510	0.685	2	0.342	0.999	0.995	0.000
	B10	1.143	0.103	11.066	^*^	0.715	0.511								
	B11	1.062	0.092	11.509	^*^	0.767	0.588								
	B12	1.114	0.103	10.860	^*^	0.696	0.484								

^*^Indicate *p* less than 0.001.

**Table 6:** Burnout competition model fitting indicators.

Patterns of second-order validation factors for burnout	χ2	df	χ2/df	GFI	AGFI	CFI	RMSEA
0.Null model	2748.931	66	41.650	0.292	0.630	0.000	0.331
1.The first-order third factor has a fully correlated model	95.925	51	1.881	0.962	0.942	0.983	0.049
2.Second order factor model	95.925	51	1.881	0.962	0.942	0.983	0.049
3.Suggested value	The smaller the better	The bigger the better	<5	>0.9	>0.9 (59)	>0.9	<0.08

#### 4.4.2. Construction of the model

This study explores the relationship between job burnout, anxiety, and depression based on a theoretical model. The model suggests that anxiety and depression can impact job burnout, and a hypothetical model was created using nurses’ anxiety as an independent variable and depression as an intermediary variable. The model fit indices, with χ2 = 342.096, df = 164, χ2/df = 2.086, GFI = 0.917, AGFI = 0.893、CFI = 0.966, RMSEA = 0.054, falls within an acceptable range, indicating a good model fitting effect (Figure 1).

#### 4.4.3. Analysis of the total, direct, and indirect effects of the model

To determine if the model had a mediation effect, both Sobel and Bootstrap methods were used. The Sobel mediation effect was verified with a Z value of 4.55, which exceeded the standard of 2, indicating a mediation effect. The Bootstrap method also confirmed a partial mediation effect. The results indicate that anxiety has a total effect of 1.413 on job burnout, with a direct effect of 0.829 and an indirect effect of 0.584 (Table 7).

**Table 7:** Analysis of the total, direct, and indirect effects of the model.

Effects	Variables	Point Estimation	Product of Coefficients		Bootstrapping					
					Bias-Corrected 95% CI			Percentile 95% CI		
			S.E.	Z	Lower	Upper	*p*	Lower	Upper	*p*
Total	Anxiety→Burnout	1.413	0.124	11.4	1.175	1.664	0.001	1.178	1.667	0.001
Direct	Anxiety→Burnout	0.829	0.167	5.0	0.517	1.184	0.001	0.501	1.167	0.001
Indirect	Anxiety→Burnout	0.584	0.150	3.9	0.312	0.915	0.001	0.313	0.917	0.001

2,000 bootstrap samples.

## 5. Discussion

The findings of the study indicate that the mean score for emotional exhaustion among participants is 22.12, indicating moderate levels of this phenomenon. Similarly, the average score for depersonalization is 9.62, suggesting high levels of this condition. Additionally, the mean score for decreased personal accomplishment is 26.70, indicating high levels of this aspect. Furthermore, the study reveals that the prevalence of severe burnout among nurses is 7.3%. The scores obtained in this study were found to be higher than the previous record of burnout among 1,621 nurses in Rizhao City before the epidemic. Specifically, the average score for emotional exhaustion was 18.14, depersonalization was 4.64, and decreased personal accomplishment was 34.59 (60). The study found that the average subdimension score of emotional exhaustion was 18.9, the average score of depersonalization subdimension was 7.3, and the average score of decreased personal accomplishment subdimension was 11.4, which was higher than another study (61). In comparison, the average subdimension score of emotional exhaustion was 26.6, the average score of depersonalization subdimension was 10.2, and the average score of decreased personal accomplishment subdimension was 27.3 in the other study (62). These results suggest that burnout scores differ across time, regions, and groups. Overall, nurses experienced higher burnout scores during the epidemic than before. This is likely due to the added pressure of the uncertain hospital environment of the new crown epidemic, as well as additional tasks such as nursing, sample collection, and out-of-home support. These factors increase the work pressure of nurses and promote the emergence of job burnout. However, a systematic review found that nurses’ burnout scores did not differ significantly before and during the pandemic (63). Consequently, both in emergencies and in normal daily routines, healthcare institutions should develop tailored occupational health programs and improve working conditions (64).

The incidence of job burnout is known to increase in poor psychological conditions. Various studies conducted both domestically and internationally have found a positive correlation between anxiety, depression, and job burnout. A cross-sectional survey of 3,527 samples found anxiety (OR = 4.87) and depression (OR = 4.06) to be risk factors for job burnout (65). One study in China have also found anxiety to be positively correlated with emotional exhaustion (r = 0.637), depersonalization (r = 0.417), and decreased personal accomplishment (r = −0.242), while depression is positively correlated with job burnout (34). This study also found a positive correlation between anxiety, depression, and job burnout (emotional exhaustion: r = 0.569, depersonalization: r = 0.406, decreased personal accomplishment: r = −0.378), but the causal relationship between the three is still unclear. To further explore the relationship between anxiety, depression, and job burnout, a structural equation model was constructed.

Previous studies have shown that anxiety and depression can both impact job burnout through other mediating variables (66, 67). Additionally, other investigations have revealed that burnout can serve as a mediating factor in the regulation of anxiety and depression (68–70). In the current research, we have developed a hypothesis positing that anxiety could potentially exert an indirect influence on burnout through the mediating factor of depression. This particular aspect of our study sets it apart from previous inquiries in the field. The findings of our study further corroborate this hypothesis. The results indicate that anxiety has a partial mediating effect on job burnout, with both direct (0.829) and indirect (0.584) effects observed. The direct effect accounts for 58.7% of the total effect, while the indirect effect accounts for 41.3%. These findings suggest that nurses who experience higher levels of anxiety are more likely to experience burnout, and that depression can exacerbate this relationship. Overall, these results highlight the importance of addressing both anxiety and depression in the prevention and management of job burnout among nurses. The comprehensive findings of the study offer substantial evidence in favor of accepting all the proposed hypotheses and their underlying assumptions.

Some questions in the scale were deleted in this study based on the modification index and square multiple correlation. The model fit could be improved based on the modification index. However, it is important to note that this correction is data-driven and may introduce coincidental errors in probability, limiting its generalizability to other samples (71). If the square multiple correlation falls below 0.6, it suggests that the corresponding topic should be removed from the analysis as it fails to adequately represent the construct characteristics (47, 48). Furthermore, it is recommended that a construct should ideally consist of 4 to 6 measurement variables (72), and all constructs in this study met this criterion by including 4 variables. Since the process of model generation is implicated in data-driven issues and there is no guarantee that the results of the model correction will be consistent with the overall results, the current study requires a new set of samples to perform the test of cross validity to assess the stability of the model (73, 74).

There are many scales used to measure different symptoms of anxiety and depression, this study used GAD-7 and PHQ-9 to measure anxiety and depression, respectively, (75). Although these two scales are classic psychological scales with high reliability and validity, the process of using the scales is only to add up the scores of the respondents to get a total score, and then determine whether there are symptoms of anxiety and depression according to the criteria. Anxiety and depression, as a complex mental illness, have many different symptoms, and the practice of grouping different symptoms into a total score cannot truly reflect the behavior and symptoms of an individual (40, 76). In addition, the current study deleted some questions in the scale to obtain a better model during model modification, a practice that improves the fit of the model but loses important information to a certain extent. Therefore, the scale can only be used as an auxiliary tool in clinical applications, and the most crucial thing is to rely on the questioning technique.

## 6. Limitations

Several limitations of the study should be settled. First, an self-assessment online questionnaire was implemented in this study, the participants will fill in the answer at random affected by response and social desirability bias. Second, we only surveyed health workers in Quanzhou, the calculated sample size could not be fully achieved, and the applicability to other regions requires further research. At last, SEM was often used to quantitatively verify relationships between variables, limiting conclusions about causality because of the cross-sectional data.

## 7. Contributions and conclusions

Our findings showed that a positive correlation between anxiety and depression on burnout, which was conform to our hypothesis. This finding contributes to understanding the relationship between burnout and mental health as well as providing additional data support for existing models. In addition, this result may also explain that during times of emergency, such as a pandemic with COVID-19 or public health emergencies, caregivers may be under more stress leading to burnout.

Nurses exhibit a greater prevalence of anxiety, depression, and burnout. Anxiety not only directly impacts burnout, but also indirectly influences burnout through its association with depression. Depression was identified as a mediating factor in the connection between anxiety and burnout. It is imperative for hospital administrators to prioritize the mental health of nurses and support them to strengthen psychological testing and counselling.

## Data availability statement

The original contributions presented in the study are included in the article/Supplementary material, further inquiries can be directed to the corresponding author.

## Ethics statement

The studies involving humans were approved by the ethical committee of Quanzhou First Hospital (NO.Quan Yi lun 2020181). The studies were conducted in accordance with the local legislation and institutional requirements. The participants provided their written informed consent to participate in this study.

## Author contributions

FL conceived and designed the experiments, performed the experiments, analyzed the data, prepared figures and/or tables, authored or reviewed drafts of the article, and approved the final draft. YZ authored or reviewed drafts of the article, and approved the final draft. YC prepared figures and/or tables and authored or reviewed drafts of the article. ZT conceived and designed the experiments, authored or reviewed drafts of the article, and approved the final draft. All authors contributed to the article and approved the submitted version.

## Figures and Tables

**Table:** 

SEM	Structural Equation Model
COVID-19	Corona Virus Disease 2019
PTSD	Post-Traumatic Stress Disorder
GAD-7	the 7-item Generalized Anxiety Disorder Questionnaire
PHQ-9	Patient Health Questionnaire-9
MBI	Maslach Burnout Inventory
KMO	Kaiser-Meyer-Olkin
SMC	Square Multiple Correlation
MI	Modification Index
CFA	Confirmatory Factor Analysis
CR	Composite Reliability
AVE	Average Variance Extracted
χ^2^	Chi-square
χ^2^/df	Normed Chi-square
GFI	Goodness of Fit Index
AGFI	Adjusted-Goodness of Fit Index
CFI	Comparative Fit Index
RMSEA	Root Mean Square Error of Approximation
