# Supplementary material for: The mediation effect analysis of nurse’s mental health status and burnout under COVID-19 epidemic
Source: Front Public Health. 2023 Oct 17;11:1221501. doi: 10.3389/fpubh.2023.1221501 (PMC10616456; doi:10.3389/fpubh.2023.1221501)
Supplement: Supplementary file 1 [file Data_Sheet_1.docx]

Supplementary Material

The Mediation Effect Analysis of Nurse's Mental Health Status and Burnout under COVID-19 Epidemic

**Fuzhi Liu^1^, Yanyan Zhao ^2^, Yangjia Chen^1^, Zhuote Tu^1^**

*** Correspondence:** Zhuote Tu, 2005016@qzmc.edu.cn

# Supplementary Tables

##### Table1. Sample Covariances

|  | **B9** | **B10** | **B11** | **B12** | **B5** | **B6** | **B7** | **B8** | **B1** | **B2** | **B3** | **B4** | **D4** | **D3** | **D2** | **D1** | **A4** | **A3** | **A2** | **A1** |
| --- | --- | --- | --- | --- | --- | --- | --- | --- | --- | --- | --- | --- | --- | --- | --- | --- | --- | --- | --- | --- |
| **B9** | 1.040 |  |  |  |  |  |  |  |  |  |  |  |  |  |  |  |  |  |  |  |
| **B10** | 0.557 | 1.218 |  |  |  |  |  |  |  |  |  |  |  |  |  |  |  |  |  |  |
| **B11** | 0.496 | 0.581 | 0.913 |  |  |  |  |  |  |  |  |  |  |  |  |  |  |  |  |  |
| **B12** | 0.535 | 0.592 | 0.572 | 1.220 |  |  |  |  |  |  |  |  |  |  |  |  |  |  |  |  |
| **B5** | 0.392 | 0.429 | 0.392 | 0.394 | 1.346 |  |  |  |  |  |  |  |  |  |  |  |  |  |  |  |
| **B6** | 0.430 | 0.407 | 0.408 | 0.406 | 1.198 | 1.515 |  |  |  |  |  |  |  |  |  |  |  |  |  |  |
| **B7** | 0.278 | 0.265 | 0.276 | 0.266 | 0.700 | 0.702 | 1.013 |  |  |  |  |  |  |  |  |  |  |  |  |  |
| **B8** | 0.464 | 0.427 | 0.395 | 0.411 | 0.753 | 0.830 | 0.487 | 1.459 |  |  |  |  |  |  |  |  |  |  |  |  |
| **B1** | 0.467 | 0.559 | 0.500 | 0.475 | 0.792 | 0.817 | 0.467 | 0.684 | 1.525 |  |  |  |  |  |  |  |  |  |  |  |
| **B2** | 0.575 | 0.664 | 0.565 | 0.608 | 0.887 | 0.925 | 0.513 | 0.774 | 1.360 | 2.151 |  |  |  |  |  |  |  |  |  |  |
| **B3** | 0.605 | 0.718 | 0.609 | 0.641 | 0.876 | 0.965 | 0.572 | 0.732 | 1.342 | 1.817 | 2.128 |  |  |  |  |  |  |  |  |  |
| **B4** | 0.527 | 0.596 | 0.533 | 0.450 | 0.910 | 0.963 | 0.518 | 0.783 | 1.087 | 1.532 | 1.399 | 2.024 |  |  |  |  |  |  |  |  |
| **D4** | 0.341 | 0.390 | 0.358 | 0.438 | 0.488 | 0.523 | 0.286 | 0.439 | 0.678 | 0.844 | 0.841 | 0.700 | 0.784 |  |  |  |  |  |  |  |
| **D3** | 0.337 | 0.367 | 0.304 | 0.366 | 0.405 | 0.466 | 0.219 | 0.399 | 0.595 | 0.720 | 0.677 | 0.583 | 0.614 | 0.868 |  |  |  |  |  |  |
| **D2** | 0.290 | 0.317 | 0.393 | 0.343 | 0.444 | 0.479 | 0.309 | 0.403 | 0.494 | 0.559 | 0.612 | 0.521 | 0.454 | 0.398 | 0.577 |  |  |  |  |  |
| **D1** | 0.338 | 0.385 | 0.369 | 0.364 | 0.443 | 0.462 | 0.255 | 0.406 | 0.644 | 0.715 | 0.706 | 0.608 | 0.594 | 0.512 | 0.418 | 0.759 |  |  |  |  |
| **A4** | 0.310 | 0.368 | 0.358 | 0.332 | 0.417 | 0.430 | 0.270 | 0.372 | 0.562 | 0.694 | 0.641 | 0.573 | 0.436 | 0.425 | 0.330 | 0.367 | 0.558 |  |  |  |
| **A3** | 0.304 | 0.293 | 0.318 | 0.284 | 0.364 | 0.366 | 0.248 | 0.292 | 0.512 | 0.551 | 0.576 | 0.491 | 0.358 | 0.349 | 0.325 | 0.361 | 0.347 | 0.500 |  |  |
| **A2** | 0.283 | 0.286 | 0.256 | 0.265 | 0.428 | 0.422 | 0.274 | 0.341 | 0.473 | 0.551 | 0.537 | 0.476 | 0.375 | 0.348 | 0.308 | 0.347 | 0.343 | 0.310 | 0.466 |  |
| **A1** | 0.246 | 0.297 | 0.294 | 0.277 | 0.384 | 0.404 | 0.254 | 0.330 | 0.516 | 0.570 | 0.584 | 0.481 | 0.398 | 0.374 | 0.333 | 0.389 | 0.376 | 0.353 | 0.337 | 0.482 |

**Supplementary Figure**


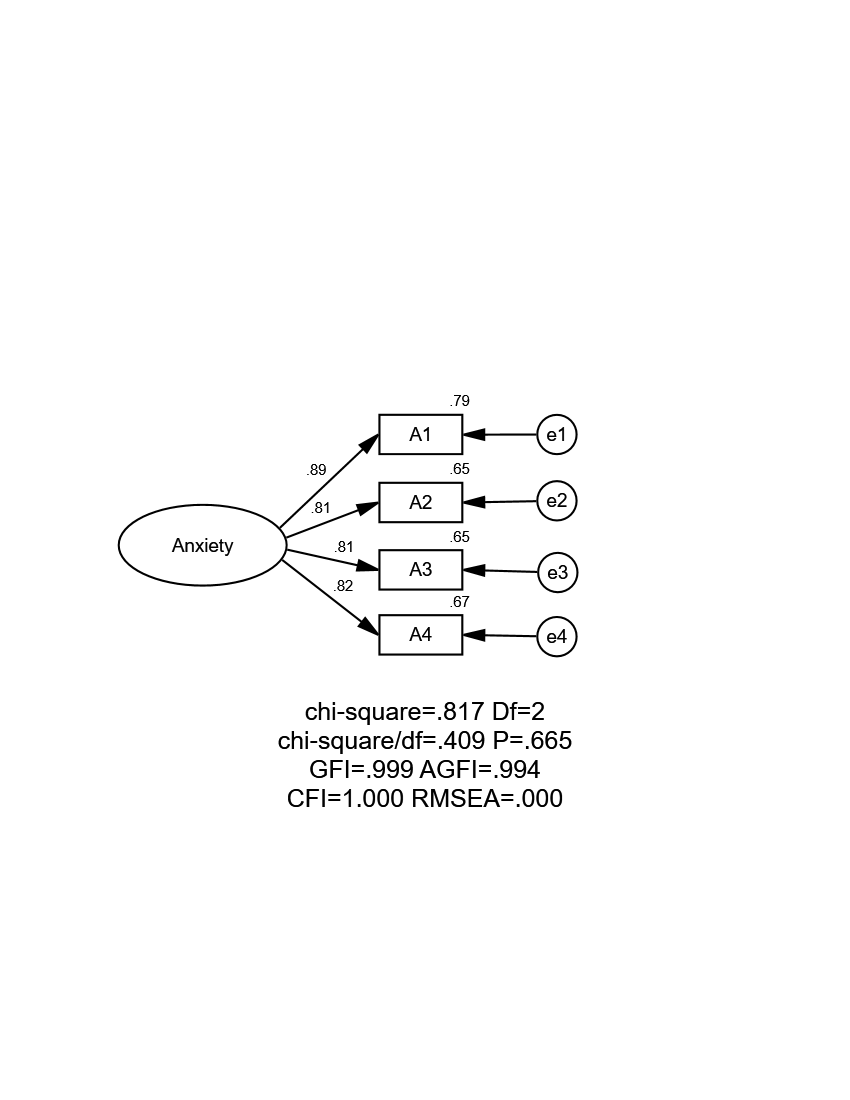

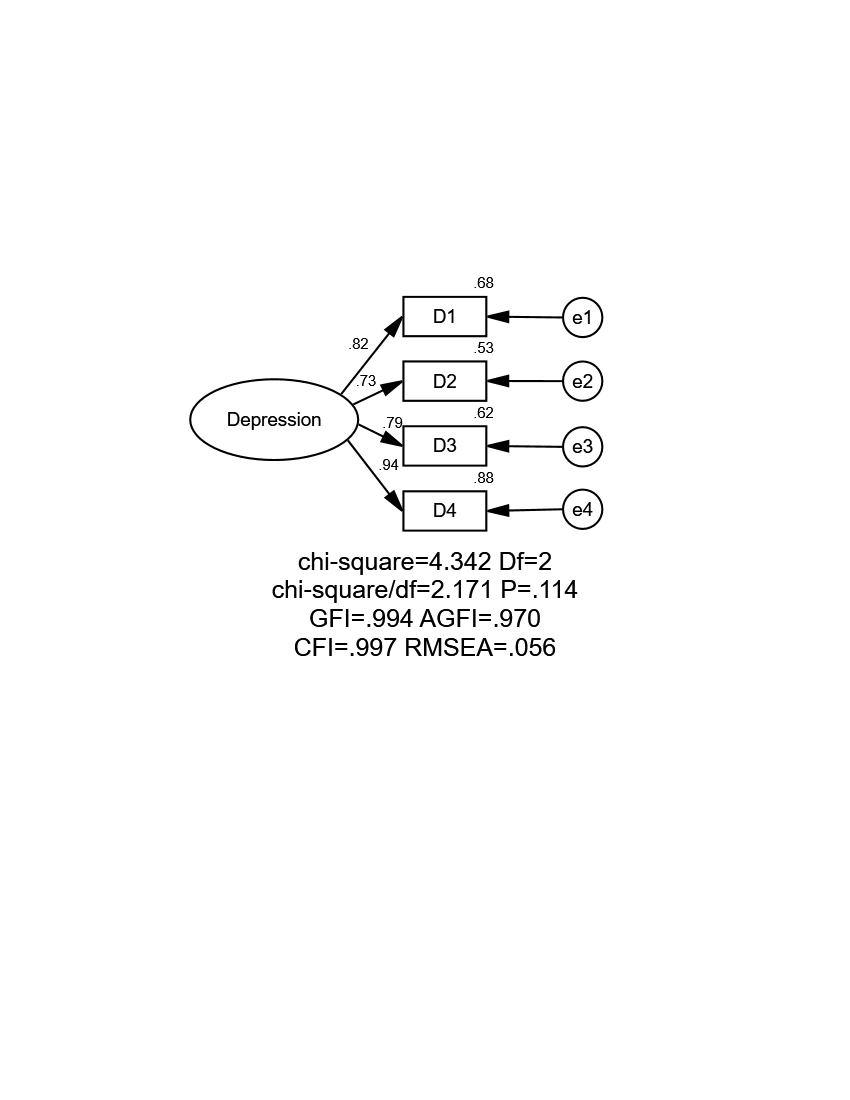


**Figure 1 Figure 2**


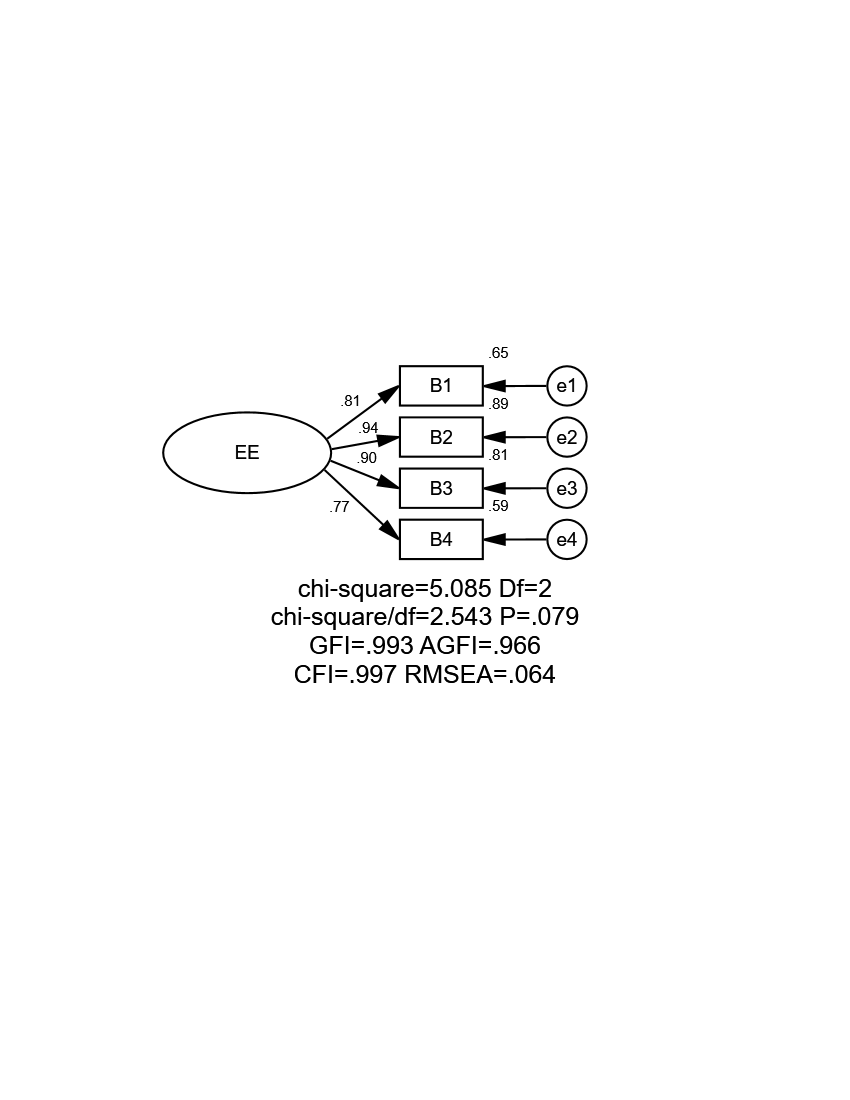

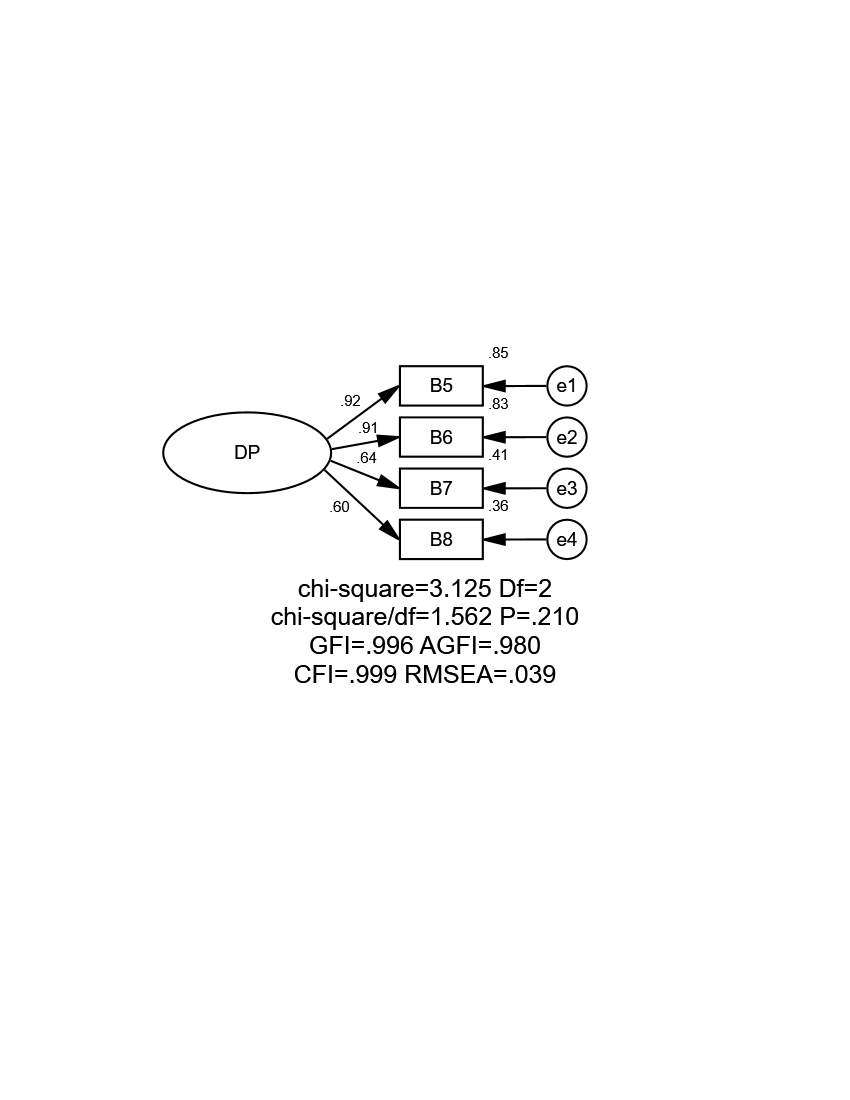

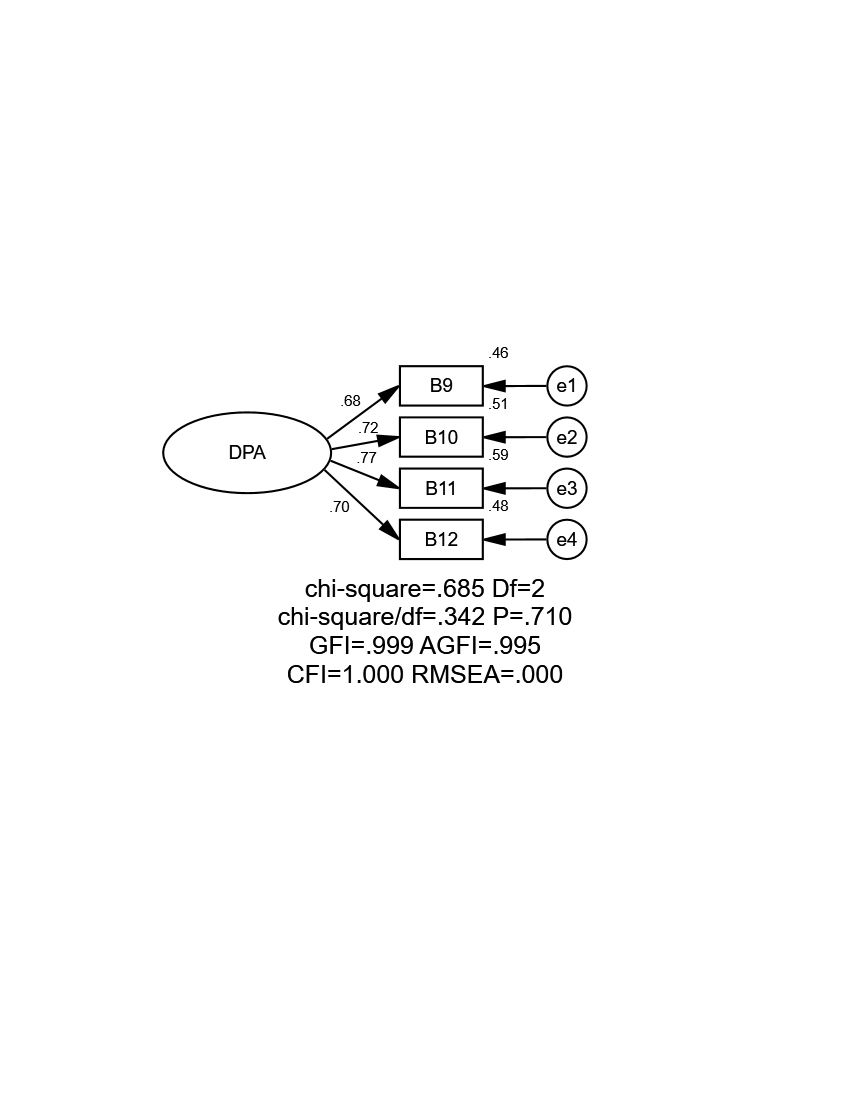


**Figure 3 Figure 4 Figure 5**

Figures 1-5: The initial test of the measurement model resulted in adequate fit to the data

Note: EE indicate emotional exhaustion; DP indicate depersonalization and DPA indicate decreased personal accomplishment
